# Corneal inflammatory cell infiltration predicts disease activity in chronic inflammatory demyelinating polyneuropathy

**DOI:** 10.1038/s41598-021-94605-7

**Published:** 2021-07-26

**Authors:** Jeremias Motte, Thomas Grüter, Anna Lena Fisse, Yesim Bulut, Zornitsa Stykova, Tineke Greiner, Elena Enax-Krumova, Min-Suk Yoon, Ralf Gold, Martin Tegenthoff, Dietrich Sturm, Kalliopi Pitarokoili

**Affiliations:** 1 Department of Neurology, St. Josef-Hospital, Ruhr-University Bochum, Gudrunstrasse 56, 44791 Bochum, Germany; 2 Immunmediated Neuropathies Biobank (INHIBIT), Ruhr-University Bochum, Bochum, Germany; 3 Department of Neurology, Bergmannsheil University Hospital, Ruhr University, Bochum, Germany; 4 Department of Neurology, Evangelisches Krankenhaus Hattingen, Hattingen, Germany; 5 Department of Neurology, Agaplesion Bethesda Krankenhaus, Wuppertal, Germany

**Keywords:** Peripheral neuropathies, Neurology, Predictive markers, Prognostic markers

## Abstract

The assessment of disease activity is fundamental in the management of chronic inflammatory demyelinating polyneuropathy (CIDP). Previous studies with small patient numbers found an increase of corneal immune cell infiltrates as a potential marker of inflammation in patients with CIDP. However, its clinical relevance remained unclear. The present study aimed to determine whether the amount of corneal inflammatory cells (CIC) measured by corneal confocal microscopy (CCM) detects disease activity in CIDP. CIC were measured in 142 CCM-investigations of 97 CIDP-patients. Data on clinical disease activity, disability (INCAT-ODSS) and need for therapy escalation at the timepoint of CCM, 3 and 6 months later were analyzed depending CIC-count. Pathological spontaneous activity during electromyography was examined as another possible biomarker for disease activity in comparison to CIC-count. An increased CIC-count at baseline was found in patients with clinical disease activity and disability progression in the following 3–6 months. An increase to more than 25 CIC/mm^2^ had a sensitivity of 0.73 and a specificity of 0.71 to detect clinical disease activity and a sensitivity of 0.77 and a specificity of 0.64 to detect disability progression (increasing INCAT-ODSS) in the following 6 months. An increase to more than 50 CIC/mm^2^ had a sensitivity of about 0.51 and a specificity of 0.91 to detect clinical disease activity and a sensitivity of 0.53 and a specificity of 0.80 to detect disability progression. CIC count is a non-invasive biomarker for the detection of disease activity in the following 6 months in CIDP.

## Introduction

Markers of disease activity are essential for adequate clinical management and therapy monitoring in chronic inflammatory demyelinating polyneuropathy (CIDP). Up to 30% of all CIDP patients require a therapy escalation due to an aggressive disease course^1^. In such patients, early therapy adjustment appears to prevent clinical disability^1–4^. Therefore, early and reliable biomarkers are essential to detect patients at risk with active disease course^5^. However, disease activity in CIDP is difficult to determine^6^. The CIDP disease activity status (CDAS) is a simple score for clinical disease activity, however this score was validated to classify the long-term outcome and response to treatment in CIDP^7^. Established methods such as nerve conduction studies (NCS) can indicate disease progression but are often delayed^8–10^. In the chronic progressive disease phase, identification of an ongoing progression in NCS can be challenging due to a high degree of axonal damage. Electromyography (EMG) can detect pathological spontaneous activity (PSA), demonstrating denervation and axonal damage^10^. Nevertheless, PSA might persist several months to years after axonal damage making it also not suitable as a dynamic biomarker. Thus, PSA can serve rather as a marker for disease progression in a long-term setting, as recently shown by our group^10^.

Corneal confocal microscopy (CCM) is a non-invasive method to image the corneal subbasal nerve plexus as well as cellular corneal elements like corneal inflammatory cells (CIC)^11–13^. It is assumed that the majority of these immunological cells are antigen presenting cells like Langerhans cells^14^. However, also other types of cell populations can be identified by morphological and biochemical features^14–17^. The number of CIC can be elevated in systemic inflammatory diseases like rheumatoid arthritis^18^. We previously reported that CIC count could be a prognostic marker for disease activity in a small cohort of 17 CIDP patients^19^. However, their clinical relevance is yet to be confirmed due to a lack of a prospective clinical follow-up assessment so far.

Aim of the present longitudinal study was to validate the CIC count in CCM as a reliable biomarker for the detection of disease activity in CIDP.

## Methods

### Patients

In total, 97 CIDP patients were recruited and prospectively analyzed regarding demographic, clinical, and treatment data during November 2015 and December 2019. All patients gave their informed consent prior to their inclusion in the study. Ethics committee of the Ruhr-University Bochum approved the study (Bio-Nerve; vote-no. 4905-14, as well as Immunmediated Neuropathies Biobank INHIBIT; vote-no. 18-6534-BR). Patients were diagnosed in accordance with the diagnostic and electrophysiological criteria of European Federation of Neurological Societies/Peripheral Nerve Society (EFNS/PNS)^20^. CIDP was further differentiated in typical and atypical CIDP in accordance to Doneddu et al.^21^.

### Study design

The study was performed as a two-center prospective observational study in the Department of Neurology, University Hospital—St. Josef-Hospital, Ruhr-University Bochum, and the Department of Neurology, BG University Hospital Bergmannsheil gGmbH, Ruhr-University Bochum, Germany.

**Figure 1:**
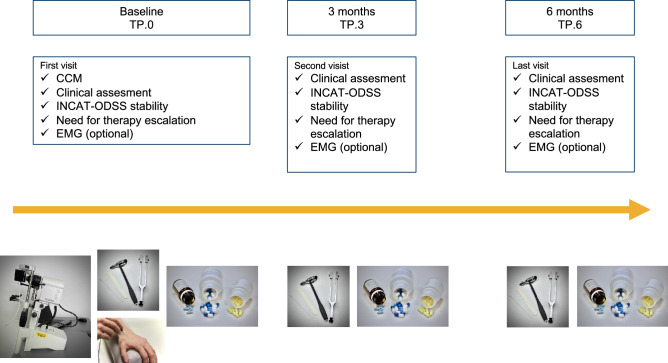
Overview of the study design. CCM was performed once at baseline, clinical status was followed up for 6 months. *TP* timepoint, *CCM* confocal corneal microscopy, *INCAT-ODSS* INCAT overall disability sum score, *EMG* electromyography.

After power analysis using G*Power (version 3.1.9.6, Heinrich-Heine-Universität Düsseldorf, Germany) resulting in 136 needed patients to depict an effect size of 0.64 with a p ≤ 0.05 we analyzed data of 142 data sets. Ninety-seven consecutive CIDP patients were prospectively included, 28 of them underwent more than one CCM examination (two examinations n = 28, three examinations n = 13, four examinations n = 4), resulting in additional 45 CCM sets. They were analyzed as separate data sets as they were more than 6 months apart from the baseline CCM. Every CCM examination was defined as at time-point zero (TP.0). Patients underwent clinical examination at TP.0 as well as three (TP.3) and six (TP.6) months after CCM. Different, blinded investigators performed CCM and clinical examination. An overview of the study design is shown in Fig. 1.

### Corneal confocal microscopy

All CCM were executed by TGre and DS using a Heidelberg Retinal Tomograph III with a Rostock Cornea Module (Heidelberg Engineering GmbH, Heidelberg, Germany) as previously described^22^. Five high-quality images of one eye were analyzed with ACC‐Metrics software, version 2.0 (Manchester, United Kingdom)^23^. CIC were counted manually by DS and reported as total cell count per mm^2^. We summarized all immunological cells in this analysis as CIC. The CCM examiners were blinded for the clinical outcome, medical history and EMG results. We analyzed, if a high CIC count at TP.0 as a marker for disease activity was associated to worse outcome at each time point (TP.0, TP.3, TP.6).

### Electromyography

Electromyographic examination as an invasive method was not performed as part of this study, but if EMG evaluation was carried out in clinical routine independently from the study on indication of the treating physician at TP.0, results were collected and were included in the analysis. Needle EMG was performed with a Dantec™ Keypoint^®^ G4 four channel electromyography device (Natus Europe GmbH, Planegg, Germany) and corresponding disposable concentric monopolar needle electrode 50 × 0.46 mm with 0.07 mm^2^ recording area (Value Line DCN, Natus, Ireland) as described in Stöhr et al^24^. PSA was sampled in the tibial anterior muscle in ten different needle locations each observed for 10 s. PSA was considered if fibrillations and positive sharp waves occurred in more than 10% of the analyzed needle positions^24^. Pathological spontaneous activity in EMG was considered as other possible biomarker for disease activity for comparison to CIC count. EMG was available in 55 cases. The presence of PSA at TP.0 was compared to disease activity at TP.0, TP.3 and TP.6 measured by the outcome parameters described below. Moreover, PSA at TP.0 was directly compared to CIC count at TP.0.

### Definition of outcome parameters

To assess the *disease activity*, three outcome parameters were obtained (definition of see section below):

1. clinical disease activity
2. worsening or stability of Inflammatory Neuropathy Cause and Treatment overall disability score (INCAT-ODSS)
3. need for therapy escalation.

#### Clinical disease activity

Expert neurologists (JM, ALF, TGrü) were blinded to CIC count and EMG and assessed the patients for disease activity based on their medical history, the clinical course and in consultation with the independent treating physicians. The disease was categorized in ‘active’ or ‘stable’.

#### INCAT-ODSS

Disability was quantified by two examiners (JM and ALF) using INCAT-ODSS as current gold standard for clinical disability of CIDP patients^25^, 3 months before TP.0, at TP.0, TP.3 and TP.6. *Stability* was defined as no change of INCAT-ODSS for 3 months before each timepoint. *Instability* was defined as an increase of at least one INCAT-ODSS point 3 months before each timepoint.

#### Need for therapy escalation

Therapy escalation was defined as:

- New application or dosage increase of a first-line treatment with intravenous immunoglobulins, corticosteroids or plasma exchange.
- New application of an immunotherapy other than first-line treatment (i.e. azathioprine, mycophenolate, cyclosporine, or rituximab).

### Statistics

Statistical analysis was performed using IBM^®^ SPSS Statistics (version 26.0.0.0). Clinical outcome parameter and CIC count were compared using Mann–Whitney test (Shapiro–Wilk test of normality was significant) and Chi-squared (χ^2^-test) for nominal variables. To measure the association of two binary variables phi coefficient (φ) was used. To determine the optimal threshold value of CIC count as disease activity marker for the following 6 months and its specificity and sensitivity, a receiver-operator-characteristic (ROC) curve was created. The area under the curve (AUC) and potential cut-off points of the CIC count were calculated. A single cut-off value was limited either to specificity or to sensitivity. To get a balanced compromise of sensitivity and specificity, two cut-off values were validated.

In case of multiple testing the Holm–Bonferroni method was used as correction. For all analyses, the statistically significant threshold was set at a p-value of < 0.05. We calculated needed effect size per sample size calculations provided by G*Power (http://www.gpower.hhu.de).

### Ethical approval

All procedures performed in studies involving human participants were in accordance with the ethical standard of the institutional and/or national research committee and with the 1964 Helsinki declaration and its later amendment or comparable ethical standards. The study was approved by the local ethics committee.

### Patient and public involvement

The patients gave spoken and written consent for publication and participation to the study.

## Results

### Clinical data

The study cohort included 97 patients, 71 male (73%) and 26 female (27%). Mean age was 56.9 years (± 13.1) and mean disease duration 4.9 years (± 4.5). The mean INCAT-ODSS at TP.0 was 3.1 (± 1.9). All 97 patients had a CIDP, 49 of them had typical CIDP (50.5%) and 48 had atypical CIDP (49.5%).

### CIC count as biomarker of disease activity

Patients who were categorized as clinically active at TP.0 and TP.3 had a significantly higher CIC count than patients who were clinically stable. At TP.6 patients with clinical activity did not have a higher CIC count than patients with stable clinical course.

**Figure 2:**
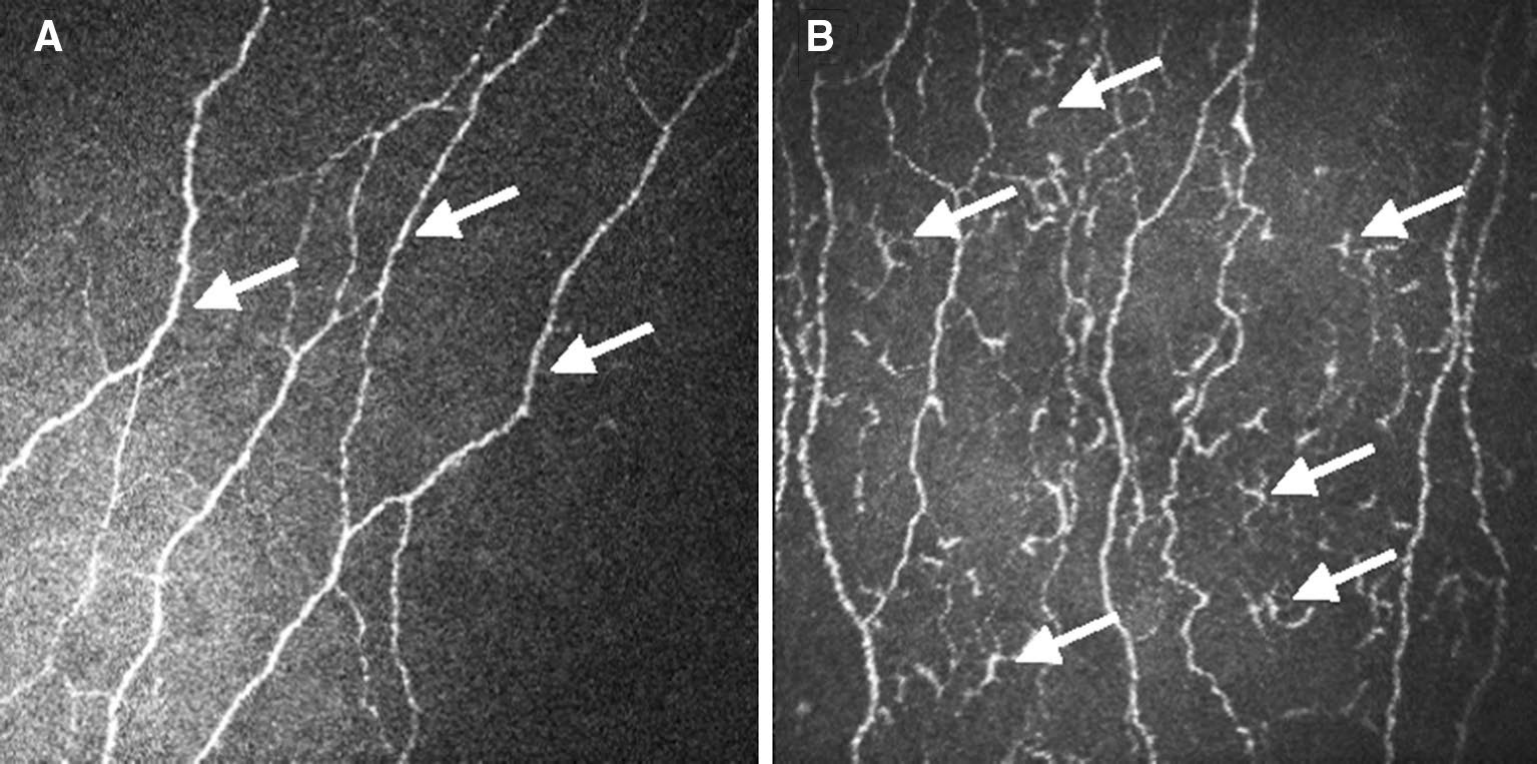
Representative CCM image of a stable patient (**A**) and an active patient (**B**). The arrows point to axons of the corneal nerves in (**A**) and to corneal inflammatory cells in (**B**). *CCM* confocal corneal microscopy.

Similarly, patients with worsening of INCAT-ODSS at TP.0 and TP.3 showed a significant higher CIC count than patients with stable INCAT-ODSS. Representative CCM images are shown in Fig. 2. For INCAT-ODSS worsening at TP.6 this association was not found.

Patients with indication for treatment escalation did not show a higher CIC count than patients without need for therapy escalation at any timepoint (Table 1).

**Table 1:** Corneal inflammatory cell count at TP.0 and on outcome in the following 6 months.

	Cell count at TP.0 median (range)		Cell count at TP.0 median (range)		Cell count at TP.0 median (range)
Clinical stable at TP.0 (n = 74)	14 (0–349)	INCAT-ODSS stable at TP.0 (n = 119)	18 (0–365)	No therapy escalation at TP.0 (n = 97)	20 (1–365)
Clinical active at TP.0 (n = 61)	54 (0–365)	INCAT-ODSS instable at TP.0 (n = 22)	54 (1–135)	Therapy escalation at TP.0 (n = 40)	28 (0–194)
p-value	0.0009	p-value	0.018	p-value	n.s.
Clinical stable at TP.3 (n = 80)	16 (1–365)	INCAT-ODSS stable at TP.3 (n = 93)	19 (0–365)	No therapy escalation at TP.3 (n = 78)	16 (0–365)
Clinical active at TP.3(n = 20)	54 (0–181)	INCAT-ODSS instable at TP.3 (n = 9)	64 (16–158)	Therapy escalation at TP.3 (n = 22)	31 (3–181)
p-value	0.016	p-value	0.016	p-value	n.s.
Clinical stable at TP.6 (n = 79)	19 (3–349)	INCAT-ODSS stable at TP.6 (n = 79)	20 (3–349)	No therapy escalation at TP.6 (n = 80)	23 (3–349)
Clinical active at TP.6 (n = 14)	32 (0–163)	INCAT-ODSS instable at TP.6 (n = 14)	32 (0–194)	Therapy escalation at TP.6 (n = 12)	32 (3–158)
p-value	n.s.	p-value	n.s.	p-value	n.s.

p-values were corrected by the Holm–Bonferroni method, groups were formed according to the outcome parameter.

The different n-values at TP.0, TP.3 and TP.6 are a result of “loss to follow-up”.

### Cut-off value, specificity and sensitivity of CIC count to detect active disease

We conducted a ROC-analysis to calculate the clinically relevant cut-off value of CIC count for detection of disease activity.

**Figure 3:**
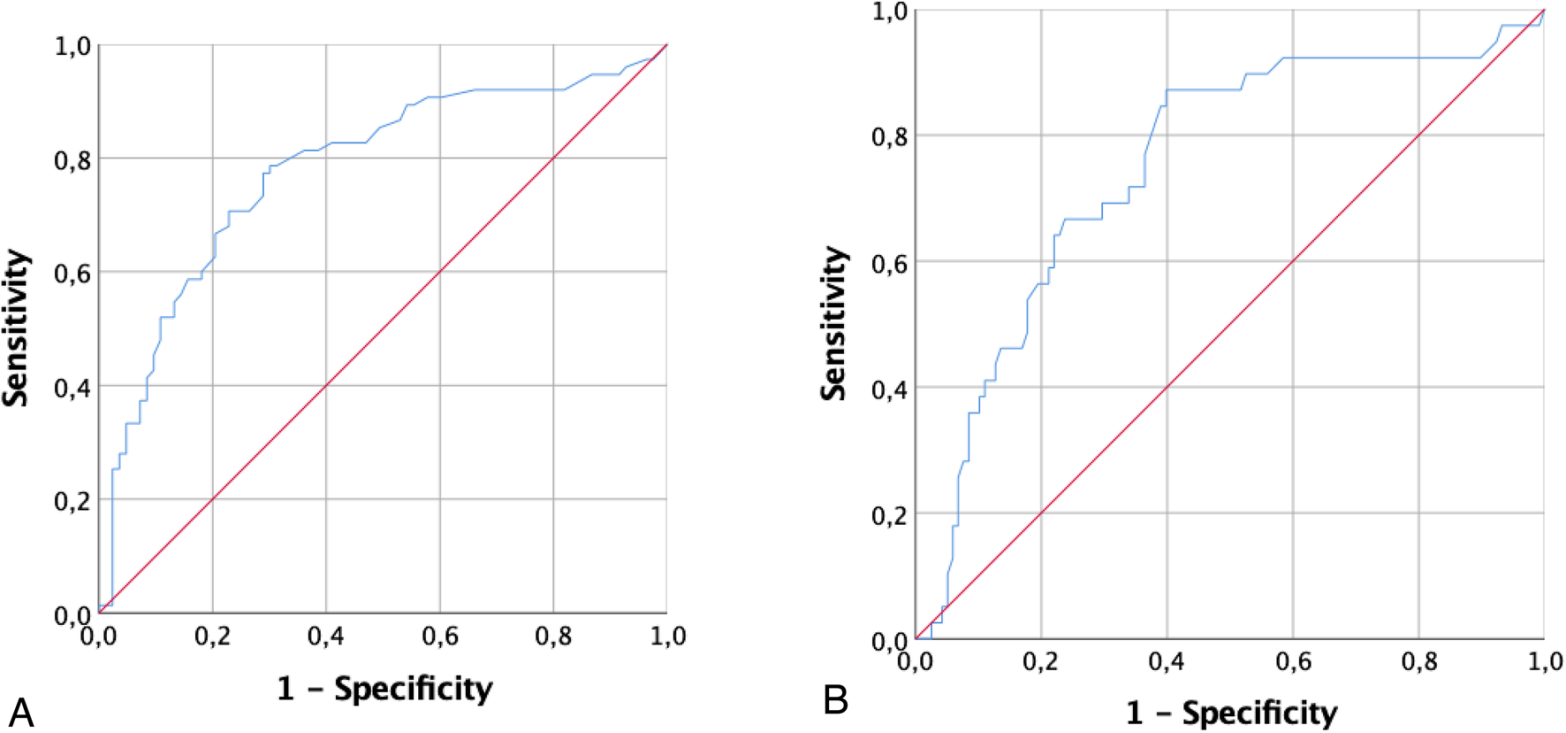
Receiver operating curves of corneal inflammatory cells to detect (**A**) clinical activity and to detect (**B**) INCAT-overall disability sum score worsening in the following 6 months.

The AUC_ROC_ to detect clinical activity at TP.0 was 0.78 (p = 0.0001, 95% CI 0.70–0.86), the AUC_ROC_ to detect INCAT-ODSS worsening in the following 6 months 0.73 (p = 0.0001, 95% CI 0.63–0.83) indicating CIC count as a fair method to detect current and early disease activity (Fig. 3A,B).

#### CIC count to detect current disease activity

A CIC count of more than 25 cells/mm^2^ detects a current disease activity with a sensitivity of 0.73 and a specificity of 0.71. Patients with a CIC count > 25 cells/mm^2^ had higher current disease activity than patients with a CIC count ≤ 25 cells/mm^2^ (χ^2^ = 35.40, p = 0.0001). Phi coefficient for the association of CIC count > 25 cells/mm^2^ and disease activity at TP.0 was φ = 0.431.

A CIC count of more than 50 cells/mm^2^ detects a current disease activity with a sensitivity of 0.51 and a specificity of 0.91. Patients with a CIC count > 50 cells/mm^2^ had higher current disease activity than patients with a CIC count ≤ 50 cells/mm^2^ (χ^2^ = 28.28, p = 0.0001). Phi coefficient for the association of CIC count > 50 cells/mm^2^ and disease activity at TP.0 was φ = 0.458.

#### CIC count to detect INCAT-ODSS worsening in the next 6 months

A CIC count of more than 25 cells/mm^2^ detects an INCAT-ODSS worsening in the next 6 months with a sensitivity of 0.77 and a specificity of 0.64. Patients with a CIC count of > 25 cells/mm^2^ more often had a worsening of INCAT-ODSS than patients with a CIC count ≤ 25 cells/mm^2^ (χ^2^ = 16.43, p = 0.0001). Phi coefficient for the association of CIC count > 25 cells/mm^2^ and disease activity at TP.0 was φ = 0.345.

A CIC count of more than 50 cells/mm^2^ detects an INCAT-ODSS worsening in the next 6 months with a sensitivity of 0.53 and a specificity of 0.80. The patients with a CIC count > 50 cells/mm^2^ more often had a worsening of INCAT-ODSS than patients with a CIC count ≤ 50 cells/mm^2^ (χ^2^ = 14.07, p = 0.0001). Phi coefficient for the association of CIC count > 50 cells/mm^2^ and disease activity at TP.0 was φ = 0.322.

#### CIC count to detect need for therapy escalation

Analyzes showed that CIC count is not suitable to detect the need of therapy escalation in the following 6 months.

### Comparison of clinical outcome parameters and CIC count with EMG

A number of 55 EMG were available at TP.0. PSA correlated with none of the outcome parameters, i.e. neither with the clinical disease activity, nor with the INCAT-ODSS worsening or the need for therapy escalation at time point of CCM (TP.0), three (TP.3) and six (TP.6) months after CCM.

Patients with PSA did not show a higher CIC count than patients without PSA (median cell count in patients with PSA 21.3/mm^2^, median cell count in patients without PSA 21.9/mm^2^, p = 0.636).

## Discussion

The main result of our study is that the amount of CIC is a noninvasive biomarker for current and short-term disease activity in CIDP, measured by clinical assessment and INCAT-ODSS. CIC count as a diagnostic test for current disease activity has a fair test quality. A CIC count of more than 25 cells/mm^2^ has a good sensitivity (77%) and a value of more than 50 cells/mm^2^ has a good specificity (80%) to detect INCAT-ODSS progression in the following 6 months.

Similar, a CIC count of more than 25 cells/mm^2^ has a good sensitivity (73%) and a value of more than 50 cells/mm^2^ has a good specificity (91%) to detect current disease activity.

We therefore suggest that patients with more than 25 cells/mm^2^ should be monitored closely clinically and a CIC count of more than 50 cells/mm^2^ should result in an expectation of a disease deterioration. When assessing disease activity with CIC a good sensitivity and specificity are important as these values means good positive and negative predictive values. This should not be misunderstood as sensitivity and specificity to diagnose a certain inflammatory neuropathy but only to detect disease activity within the group of CIDP. CIC can be compared to leukocytes, which are not specific for infectious diseases but could still be used as an activity marker in systemic infections. Whether CIC is increased only on inflammatory neuropathies or in polyneuropathies with inflammatory activity remains to be evaluated in studies with multiple groups of patients with different polyneuropathies and controls. Our data implicate that patients with risk of clinical deterioration can be identified early by measuring CIC count. That is important because early therapy escalation in inflammatory neuropathies has been described as beneficial^4,5^. However, CCM does not allow therapeutic decisions based solely on the CIC count. Only a bundle of different diagnostic methods describes the individual risk of disease activity in CIDP.

In contrast to CIC infiltration, PSA in EMG represents axonal damage. We have recently reported about PSA as a prognostic marker for CIDP patients in a long-term cohort study^10^. PSA is suitable to predict long-term clinical progression beyond 7 months^10^. We suppose that CIC infiltration represents an “active state” of inflammation, as it correlates to current disease activity, whereas over time, the correlation of CIC count and disease activity decreases. Therefore, CIC count is a marker for acute disease activity, while PSA as a sign of axonal damage has a long-term prognostic relevance. The corneal nerve fiber parameters might be a marker of axonal damage as well, but were not analyzed in this study since the longitudinal period of evaluation was very short and this parameters did not change (data not shown). Corneal nerve fiber reduction was shown in several types of neuropathy including immune-mediated neuropathies^26–28^, however acute upcoming disease activity was not analyzed in these studies. Taken together, both information, acute cell infiltration and axonal damage, are important but different aspects of the disease^4^.

Previous studies documented an increase of CIC count in CIDP compared to healthy controls^26,27^. One of these studies showed a relation between the clinical phenotype of CIDP and the number of CIC: patients with predominant motoric symptoms had the highest numbers of CIC. Besides, a direct contact of the CIC to corneal axons were described in inflammatory neuropathies^22^. We did not analyze these clinical and morphological features, which is a limitation of this study. Further histological in vivo assessment of the corneal subbasal plexus and different cell populations in animal models of autoimmune neuropathies might help to clarify the pathophysiological role of CIC.

We did not find an association between CIC count and therapy escalation. The main reason for that probably is, that escalation of therapy occurs delayed and therefore was not recorded in the 6 months follow-up period. Escalation therapy for example with cyclophosphamide or rituximab are off-label and are used cautiously which could lead to a delayed decision. A therapy escalation in CIDP has not yet been investigated in controlled studies, but only in retrospective or uncontrolled trails.

The CDAS-score^7^ is an assessment tool for clinical disease activity and was validated for the long-term outcome and response to treatment in CIDP. In this study we did not use this score as outcome parameter as we want examinate a short-term outcome parameter. However, an evaluation of the CIC as inflammatory marker and PSA as axonal marker in relation to the CDAS is a useful approach for register studies that examine long-term outcome of CIDP.

This study has three important limitations. First, it was carried out at only two tertiary centers potentially including patients with complex medical history. However, the CCM examination was carried out and analyzed blinded and independent from decisions regarding patients’ treatment. Second, there is no control group with healthy volunteers in the study protocol. However, in this pilot study we focused on short-term follow up analysis of intraindividual changes. This study did not show that the CIC is different between active CIDP and healthy controls, but between active and stable CIDP patients. Previous studies show that CIC is lower in healthy controls^26,29–31^. Last, several ophthalmologic and metabolic diseases may also influence the amount of CIC or the subbasal plexus^17,32–34^. Nevertheless, serious ophthalmic diseases were always enquired before CCM examination.

In conclusion, measurement of the CIC count is a non-invasive biomarker for assessing the disease activity in CIDP patients. CIC count detects current and future disease activity in a period up to 6 months. Therefore, CCM should be used more frequently for monitoring CIDP patients. Further studies are necessary to understand the pathophysiology behind CIC infiltration in inflammatory neuropathies.

## Data Availability

Data collected from this study are available by emailing jeremias.motte@rub.de.

## References

[1] Yoon M-S, Chan A, Gold R (2011). Standard and escalating treatment of chronic inflammatory demyelinating polyradiculoneuropathy. Ther. Adv. Neurol. Disord..

[2] Dalakas MC (2011). Advances in the diagnosis, pathogenesis and treatment of CIDP. Nat. Rev. Neurol..

[3] Ellrichmann G, Gold R, Ayzenberg I, Yoon M-S, Schneider-Gold C (2017). Two years’ long-term follow up in chronic inflammatory demyelinating polyradiculoneuropathy: Efficacy of intravenous immunoglobulin treatment. Ther. Adv. Neurol. Disord..

[4] Motte J (2021). Treatment response to cyclophosphamide, rituximab, and bortezomib in chronic immune-mediated sensorimotor neuropathies: A retrospective cohort study. Ther. Adv. Neurol. Disord..

[5] Fisse AL (2020). Comprehensive approaches for diagnosis, monitoring and treatment of chronic inflammatory demyelinating polyneuropathy. Neurol. Res. Pract..

[6] Lehmann HC, Burke D, Kuwabara S (2019). Chronic inflammatory demyelinating polyneuropathy: Update on diagnosis, immunopathogenesis and treatment. J. Neurol. Neurosurg. Psychiatry.

[7] Albulaihe H (2016). Disease activity in chronic inflammatory demyelinating polyneuropathy. J. Neurol. Sci..

[8] Décard BF, Pham M, Grimm A (2018). Ultrasound and MRI of nerves for monitoring disease activity and treatment effects in chronic dysimmune neuropathies—Current concepts and future directions. Clin. Neurophysiol..

[9] Allen JA, Lewis RA (2015). CIDP diagnostic pitfalls and perception of treatment benefit. Neurology.

[10] Grüter T (2020). Pathological spontaneous activity as a prognostic marker in chronic inflammatory demyelinating polyneuropathy. Eur. J. Neurol..

[11] Wang EF, Misra SL, Patel DV (2015). In vivo confocal microscopy of the human cornea in the assessment of peripheral neuropathy and systemic diseases. Biomed. Res. Int..

[12] Zhivov A, Stave J, Vollmar B, Guthoff R (2007). In vivo confocal microscopic evaluation of Langerhans cell density and distribution in the corneal epithelium of healthy volunteers and contact lens wearers. Cornea.

[13] Choi EY (2017). Langerhans cells prevent subbasal nerve damage and upregulate neurotrophic factors in dry eye disease. PLoS One.

[14] Gillette TE, Chandler JW, Greiner JV (1982). Langerhans cells of the ocular surface. Ophthalmology.

[15] Mayer WJ (2007). Characterization of antigen-presenting cells in fresh and cultured human corneas using novel dendritic cell markers. Investig. Ophthalmol. Vis. Sci..

[16] Vantrappen L, Geboes K, Missotten L, Maudgal PC, Desmet V (1985). Lymphocytes and Langerhans cells in the normal human cornea. Investig. Ophthalmol. Vis. Sci..

[17] Postole AS, Knoll AB, Auffarth GU, Mackensen F (2016). In vivo confocal microscopy of inflammatory cells in the corneal subbasal nerve plexus in patients with different subtypes of anterior uveitis. Br. J. Ophthalmol..

[18] Marsovszky L (2012). In vivo confocal microscopic evaluation of corneal Langerhans cell density, and distribution and evaluation of dry eye in rheumatoid arthritis. Innate Immun..

[19] Pitarokoili K (2019). Neuroimaging markers of clinical progression in chronic inflammatory demyelinating polyradiculoneuropathy. Ther. Adv. Neurol. Disord..

[20] den Bergh PYKV (2010). European Federation of Neurological Societies/Peripheral Nerve Society Guideline on management of chronic inflammatory demyelinating polyradiculoneuropathy: Report of a joint task force of the European Federation of Neurological Societies and the Peripheral Nerve Society. Eur. J. Neurol..

[21] Doneddu PE (2019). Atypical CIDP: Diagnostic criteria, progression and treatment response. Data from the Italian CIDP Database. J. Neurol. Neurosurg. Psychiatry.

[22] Petropoulos IN (2013). Repeatability of in vivo corneal confocal microscopy to quantify corneal nerve morphology. Cornea.

[23] Chen X (2016). An automatic tool for quantification of nerve fibers in corneal confocal microscopy images. IEEE Trans. Bio-med. Eng..

[24] Stöhr M, Pfister R (2014). Klinische Elektromyographie und Neurographie—Lehrbuch und Atlas.

[25] Merkies ISJ (2002). Clinimetric evaluation of a new overall disability scale in immune mediated polyneuropathies. J. Neurol. Neurosurg. Psychiatry.

[26] Stettner M (2015). Corneal confocal microscopy in chronic inflammatory demyelinating polyneuropathy. Ann. Clin. Transl. Neurol..

[27] Schneider C (2014). Corneal confocal microscopy detects small fiber damage in chronic inflammatory demyelinating polyneuropathy (CIDP). J. Peripher. Nerv. Syst..

[28] Fleischer M (2021). Corneal confocal microscopy differentiates inflammatory from diabetic neuropathy. J. Neuroinflamm..

[29] Bitirgen G, Turkmen K, Malik RA, Ozkagnici A, Zengin N (2018). Corneal confocal microscopy detects corneal nerve damage and increased dendritic cells in Fabry disease. Sci. Rep. U.K..

[30] Bitirgen G, Akpinar Z, Malik RA, Ozkagnici A (2017). Use of corneal confocal microscopy to detect corneal nerve loss and increased dendritic cells in patients with multiple sclerosis. JAMA Ophthalmol..

[31] Mobeen R (2019). Corneal epithelial dendritic cell density in the healthy human cornea: A meta-analysis of in-vivo confocal microscopy data. Ocular Surf..

[32] Lagali NS (2018). Dendritic cell maturation in the corneal epithelium with onset of type 2 diabetes is associated with tumor necrosis factor receptor superfamily member 9. Sci. Rep. U.K..

[33] Tavakoli M, Boulton AJM, Efron N, Malik RA (2011). Increased Langerhan cell density and corneal nerve damage in diabetic patients: Role of immune mechanisms in human diabetic neuropathy. Contact Lens Anterior Eye.

[34] Alzahrani Y, Colorado LH, Pritchard N, Efron N (2017). Longitudinal changes in Langerhans cell density of the cornea and conjunctiva in contact lens-induced dry eye. Clin. Exp. Optom..

